# Hyposulphite of Soda-Poisoning1Read before the annual meeting of the Iowa and Nebraska Veterinary Medical Association, November 20, 1900.

**Published:** 1901-07

**Authors:** J. G. Parslow

**Affiliations:** Shenandoah, Iowa


### REPORTS OF CASES.

### HYPOSULPHITE OF SODA-POISONING.[1]

By J. G. Parslow, V.S.,
SHENANDOAH, IOWA.

On March 19th I was called to Red Oak, Iowa, to investigate a trouble existing in a large barn of feeding horses. On my arrival I learned two had already died, and I was shown four or five that were quite sick. The symptoms, as the owner and caretaker noticed, were loss of appetite, whisking of tail, followed by light colicky pains and profuse diarrhœa, the colic gradually increasing in severity until extreme agony was evinced in the two that had died.

I examined the remainder, and found the heart action and respiration very little interfered with. Temperature from 101° to 102°. Recognizing it as a dietetic trouble, I proceeded to examine the diet. The grains, hay, and water to all appearances were good. I was then shown a keg of soda hyposulphite, and was told that each horse was receiving about a tablespoonful each day. This did not strike me at first as of any importance, but I was inquisitive enough to ask if those horses would eat soda in the coarse crystal form in their feed. The fact was then shown that the soda for the entire stable was placed in a tank and water drawn on it. When dissolved the horses were let out one at a time to water. Some would refuse to drink, while others drank heartily.

My suspicions were aroused, and I set about weighing the soda and measuring the water. This done, the result was that each gallon of water was found to contain approximately eighteen and one-half drachms of soda. This was offered after each morning's feed. Figuring on four to six gallons to those horses that did drink heartily, they were receiving from four and one-half to seven ounces of soda. I could not say that that was sufficient to kill a good, healthy horse, so I resorted to a post-mortem on the one that had died on the night previous.

The deviations from normal as I noticed were as follows :

Large intestines: totally devoid of blood, dark drab or slate color, the wall rather thicker than normal, caused from submucous

[1] Read before the annual meeting of the Iowa and Nebraska Veterinary Medical Association, November 20, 1900.

effusion. This effusion was like a jelly in consistency, yellowish in color, tinged with dark drab.

Stomach: tissues normal, but contents heavily coated with black, tarry substance where it came in contact with the walls of the organ.

Spleen: totally devoid of blood, and on section it presented a beautiful dark cherry-red; stroma, tough with a withered feeling.

Kidneys: mottled with dark-blue spots. Over each spot the capsule was markedly indentated. Underneath each spot a congested spot passed down through the cortex.

Bladder: empty, and heavily coated on lining with a milky-like secretion.

Blood: was a beautiful dark cherry color, and feebly coagulated.

This completed the post-mortem, and I announced my findings to be that of hyposulphite soda-poisoning.

*Treatment.* Remove the cause. To the worst cases I gave oleum lini, one pint, followed by light stimulants, with orders for a light laxative diet. No further loss was experienced. One or two of the worst cases convalesced slowly.

